# Camouflage of Severe Skeletal Class II Gummy Smile Patient Treated Nonsurgically with Mini Implants

**DOI:** 10.1155/2014/382367

**Published:** 2014-12-07

**Authors:** Irfan Qamruddin, Fazal Shahid, Mohammad Khursheed Alam, Wafa Zehra Jamal

**Affiliations:** ^1^Orthodontic Department, Baqai Medical University, Karachi 74600, Pakistan; ^2^Orthodontic Unit, School of Dental Science, Universiti Sains Malaysia, Kubang Kerian, 16150 Kota Bharu, Kelantan, Malaysia; ^3^Dow University of Health Science, Karachi, Pakistan

## Abstract

Skeletal class II has always been a challenge in orthodontics and often needs assistance of surgical orthodontics in nongrowing patients when it presents with severe discrepancy. Difficulty increases more when vertical dysplasia is also associated with sagittal discrepancy. The advent of mini implants in orthodontics has broadened the spectrum of camouflage treatment. This case report presents a 16-year-old nongrowing girl with severe class II because of retrognathic mandible, and anterior dentoalveolar protrusion sagittally and vertically resulted in severe overjet of 13 mm and excessive display of incisors and gums. Both maxillary central incisors were trimmed by general practitioner few years back to reduce visibility. Treatment involved use of micro implant for retraction and intrusion of anterior maxillary dentoalveolar segment while lower incisors were proclined to obtain normal overjet, and overbite and pleasing soft tissue profile. Smile esthetics was further improved with composite restoration of incisal edges of both central incisors.

## 1. Introduction

The most common reason to approach an orthodontist is esthetic concern which is compromised by malocclusion [1]. Malocclusion, which can be skeletal or dental in origin [2], is present in every society but with variable prevalence [3–5]. Class II div 1 is the most prevalent malocclusion in Pakistani population [6]. Depending on the severity, class II div 1 not only causes esthetic and functional problems but also results in psychological disturbances [7]. The treatment of class II involves growth modification in growing patients and camouflage in adults, if the skeletal discrepancy is mild to moderate. Complexity of treatment increases with the severity of sagittal discrepancy particularly when it coexists with maxillary vertical excess [8].

Maxillary vertical excess, which also can be skeletal or dentoalveolar type, presents with excessive visibility of upper incisors and excessive display of gingiva on smiling (gummy smile) [9]. More than 4 mm of gingival display is considered excessive and unattractive by patients and also by general dentist [10]. Irrespective of the cause, gummy smiles are rarely corrected with conventional mechanics and often orthognathic surgery is recommended [10]. However skeletal anchorage system has now widened the spectrum of orthodontics and is also very well accepted by patients [11, 12]. Mini screws can provide maximum anchorage to retract and intrude dentoalveolar segment simultaneously.

The following case is a severe skeletal class II with anterior maxillary dentoalveolar extrusion, which was treated with orthodontic camouflage rather than orthognathic surgery.

## 2. Case Report

A 16-year-old female patient came to the Orthodontic Department of Baqai Medical University with the presenting complaint of protrusion along with excessive visibility of upper incisors and excessive display of gums on smiling. There was no significant medical history while dental history revealed her visit to a local general dentist 2 years ago with the same complaint where she was treated by trimming of her incisors to reduce visibility.

### 2.1. Findings

**Figure 1:**
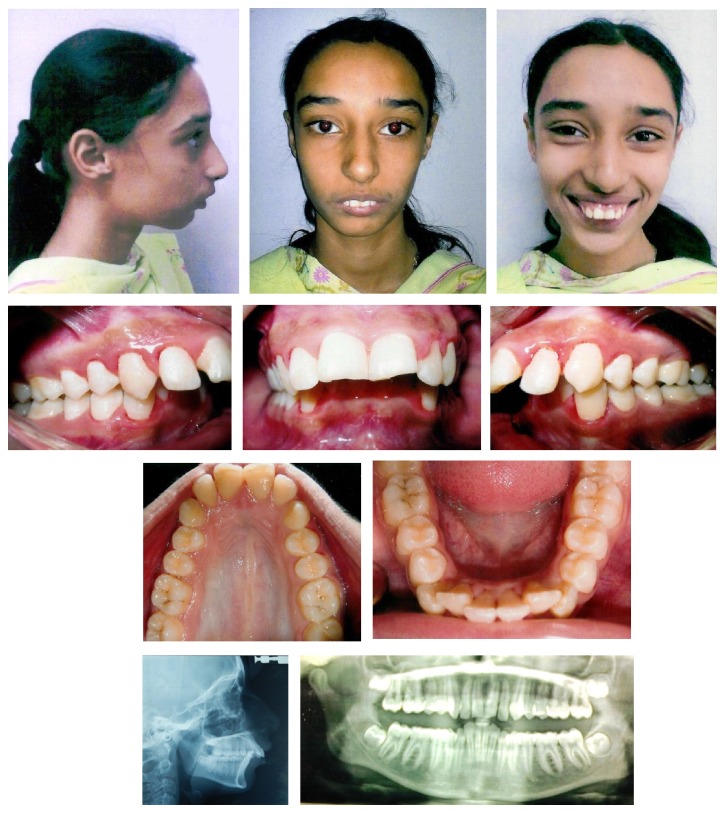
Pretreatment photographs.

Extraoral examination displayed a convex profile with mandibular deficiency and slight maxillary protrusion. Nasolabial and mentolabial sulcus were acute. Lips were incompetent, with incisor visibility of 7 mm with relaxed lips and gingival display of 6 mm on smiling commonly known as “gummy smile.” Intraoral examination revealed full cusp class II molar and class II canine relationship on both sides. The maxillary arch was elliptical in shape with mild spacing while the mandibular arch was square shaped which also showed 7 mm crowding in the anterior region. A 100% deep bite and an overjet of 13 mm were noted. Both the maxillary and mandibular midlines were coinciding with the facial midline. Oral hygiene was poorly maintained which had resulted in gingivitis (Figure 1). Temporomandibular joint evaluation revealed no signs of dislocation, malfunction, clicks, or crepitus, and the facial and masticatory muscles were asymptomatic.

Panoramic radiograph revealed no missing teeth and no sign of root resorption. The maxillary and mandibular third molars were in the formative stages. No caries or periapical lesion was visible.

Lateral cephalometric analysis showed a skeletal class II relationship with severe mandibular deficiency. Vertical analysis depicted mild hyperdivergence and steep mandibular plane angle. Upper incisors were proclined and extruded beyond the normative mean (Table 1).

### 2.2. Diagnosis and Treatment Objectives

The patient was diagnosed to have severe skeletal class II relationship with mandibular deficiency. Dental relationship was Angle's class II div 1 with anterior maxillary dentoalveolar protrusion in both sagittal and vertical planes which resulted in excessive overjet, overbite, and gummy smile. The desired treatment objectives included (1) intrusion and retraction of upper incisors to attain normal overjet and overbite with competency of lips and esthetically pleasing smile and (2) restoration of trimmed maxillary incisors.

### 2.3. Ideal and Alternate Treatment Plan

Ideal treatment plan offered to the patient was the subapical segmental osteotomy in upper jaw to move the whole anterior maxillary segment upward and backward with surgical mandibular advancement in lower jaw. To execute that plan all first premolars in both jaws were to be extracted bilaterally to decompensate the arches so that the case could be finished in class I molar and canine relationship. However the patient rejected the surgical plan; therefore alternate treatment plan was followed.

Objective of alternate treatment plan was extraction of maxillary first premolars with intrusion and retraction of upper anterior segment and nonextraction treatment in lower arch. This will finish the case in class II molar and class I canine relationship.

### 2.4. Treatment Progress

**Figure 2:**
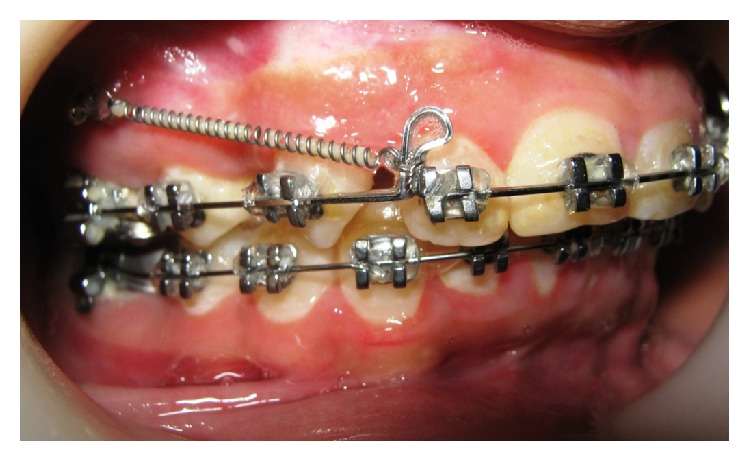
Treatment progress.

The treatment was started with banding and bonding procedure using 0.022 slot preadjusted edgewise brackets, MBT prescription. Vertical placement of brackets on central and lateral incisors was kept at the same level so that the incisal edges can be restored after the treatment. Alignment and leveling were achieved with continuous archwire used in the following sequence: 0.012 Niti, 0.016 Niti, 0.017 × 0.025 Niti followed by 0.017 × 0.025-in SS wire. Extractions of upper first premolars were carried out along with insertion of mini implant in the same appointment. Self-drilling type of titanium mini implants (1.4 mm diameter and 8 mm length) was inserted between the roots of upper first molar and second premolar bilaterally. Implants were loaded immediately with elastomeric chain to retract the canine first into class I relation. After achieving class I cuspid relationship bilaterally, NiTi closed coil springs were extended from implants up to the helix formed in the archwire distal to the lateral incisors on both sides. Force of 150 gm was applied (measured with Dentus gauge) with the force vector passing above the CRes of maxilla, so that the anterior teeth are retracted upward and backward (Figure 2). Forces were repeated after every three weeks till the extraction spaces are completely closed. Fixed appliance was removed after 27 months and the patient was referred for the restoration of central incisors. After composite restorations, acrylic retainer was given in upper arch and fixed retainer in lower arch.

## 3. Results

**Figure 3:**
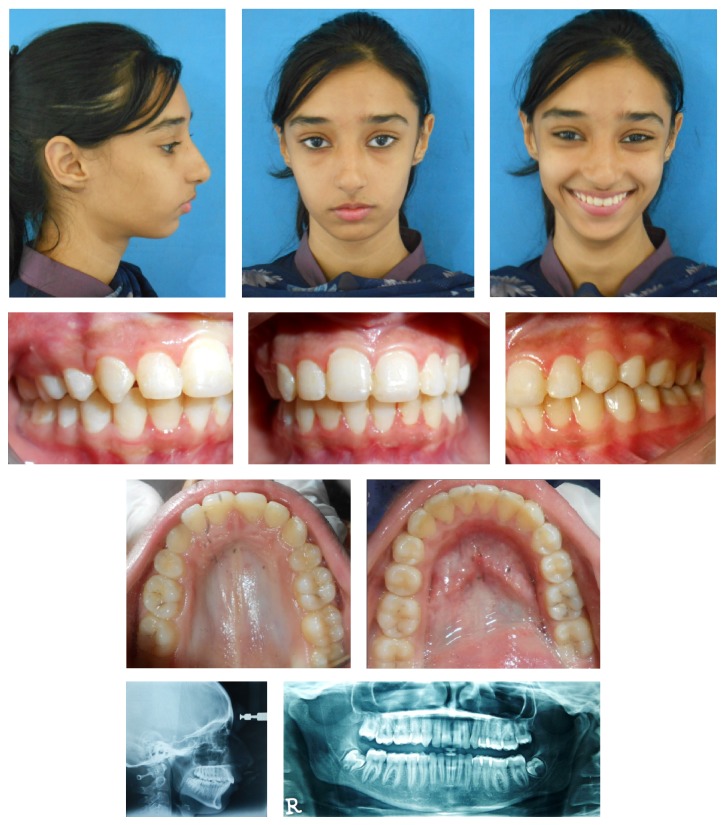
Posttreatment photographs.

Remarkable improvement in facial and smile esthetics was accomplished. Patient had competent lips and the visibility of incisors was reduced to 3 mm after restoring the incisal edges with composite filling. Smile was broader; smile arc was consonant with 1 mm gingival exposure on lateral incisors. Facial convexity was also reduced with the retraction of upper lip and mild autorotation of lower jaw in anticlockwise direction. Nasolabial angle and mentolabial sulcus were improved (Figure 3).

**Figure 4:**
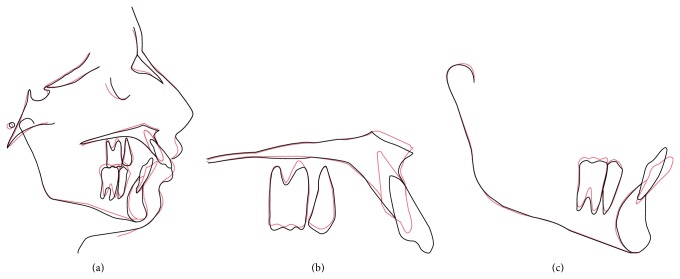
Superimposition of pretreatment (black) and posttreatment (red) cephalometric tracings (a) registered on sella with best fit on anterior cranial base; (b) maxillary composite superimposed on palatal curvature with best fit on maxillary bony structure; (c) mandibular composite registered on internal cortical outline of the symphysis.

Maxillary incisors were retracted by 6 mm whereas intrusion attained was 5 mm. Anterior dentoalveolar height was reduced by 5 mm while lower anterior dentoalveolar height was reduced by 4 mm. Lower incisors were proclined by 7° which also reduced the overjet to 2 mm and overbite to 20% (Figure 4).

## 4. Discussion

Orthognathic surgery is the only ideal treatment when there is severe skeletal discrepancy in adult patient; however, in many societies, surgery is only pursued when there is life threatening condition [13]. Surgical orthodontics is barely accepted by patients for esthetics because of multiple reasons that include financial constraints, fear of procedure, and adverse effects and also on religious grounds [13, 14]. Our patient also refused the surgical option for all the above-mentioned reasons. The other option for skeletal malocclusion is dental camouflage which involves repositioning of dentoalveolar structure to disguise the severity of skeletal problem [15]. Class II cases demand either camouflage with extractions of two maxillary and two mandibular premolars or extractions of only upper first premolars when there is no arch length discrepancy in lower arch [7, 16].

In this case upper first premolars were extracted bilaterally to retract the anterior maxillary arch and bring canines into class I occlusion. Although the patient had crowding as well as very deep curve of spee in lower arch, even then nonextraction treatment was planned in mandibular arch. The reason was the severity of skeletal discrepancy accompanying severe overjet, which was not possible to correct without mandibular teeth advancement.

Our patient also had excessive incisor and gingival display due to extrusion of anterior maxillary dentoalveolar segment, which also resulted in 100% deep bite. Posterior vertical relations including posterior maxillary and mandibular dentoalveolar heights and mandibular plane angle were close to normal. Before the advent of micro implants in orthodontics, conventional mechanics to correct deep bite always resulted in extrusion of posterior teeth [9] and concomitant clockwise rotation of mandible aggravating the class II and receding the chin more [17]. Segmental mechanics by Burstone [18] and three-piece arch by Shroff et al. [19] are an option but both mechanics are indeterminate and anchorage loss may associate. The benefits of using mini implants in this case were twofold:

- they provided maximum anchorage to retract maxillary anterior segment;
- simultaneous retraction and intrusion were possible.

**Figure 5:**
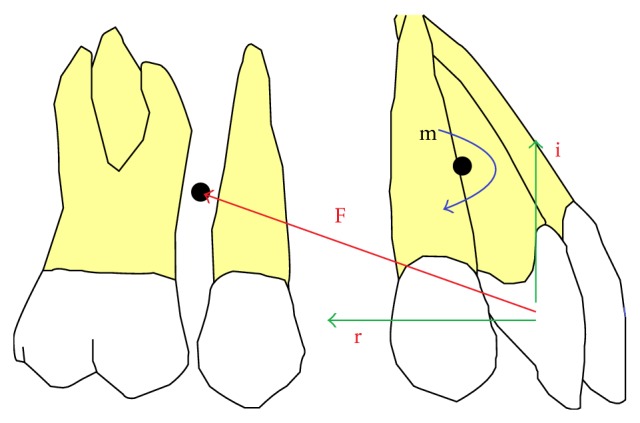
Biomechanics of force delivery system involved. F: force applied; r: retraction component; i: intrusive component; m: moment created on anterior teeth.

Occlusogingival position of mini implant determines the biomechanical effects of the force system. Applied force in this case had two components: horizontal and vertical. Horizontal component resulted in retraction (r) while vertical component moved the anterior teeth upward (i). However the force vector passed below the center of resistance of anterior teeth; therefore moment was created which also tipped the incisors lingually (Figure 5). Therefore the retraction of incisors in this case involved both the translation and tipping movement, as the inclination of the incisors was improved along with the lingual movement of roots.

Soft tissue esthetics is of utmost importance in treatment planning and overretraction of incisors can have undesirable effects; therefore overjet and overbite reduction also involved movement of lower incisors forward and downward. This also helped in flattening of curve of spee, though the intrusion was relative in lower arch.

Intrusion of posterior teeth in upper arch was not planned but superimposition of the lateral cephalometric tracings shows some intrusion of upper molars that resulted in anticlockwise rotation of lower jaw and slight improvement in chin prominence. This finding is supported by Upadhyay who also reported intrusion of upper molars in three patients while performing space closure with mini implants [17]. This movement was explained as a result of binding of archwire with the brackets and buccal tubes at later stages of incisor's intrusion [17].

There was no major significant change observed in the cephalometric skeletal measurements and the patient remained skeletally class II; however special consideration was given to the soft tissue profile and smile arc of the patient, which was further improved with restoration of incisal edges with composite after debonding.

## 5. Conclusion

Surgical orthodontics is not a very common and acceptable procedure; however use of skeletal anchorage system has broadened the horizon of camouflage treatment in moderate to severe skeletal dysplasia. Simultaneous intrusion and retraction of anterior teeth are now possible with mini implants without losing anchorage and vertical control.

## Figures and Tables

**Table 1:** Performed cephalometric measurements.

Measurements	Pretreatment	Posttreatment
SNA	80°	80°
SNB	70°	71°
ANB	10°	09°
SNMP	37°	36°
FHMP	26°	25°
MMA	24°	23°
UI-SN	104°	97°
UI-FH	115°	107°
UI-PP	117°	109°
IMPA	96°	103°
UPDH	19 mm	17 mm
UADH	29 mm	24 mm
LPDH	27 mm	28 mm
LADH	41 mm	37 mm
Nasolabial angle	80°	110°
